# Comparative effectiveness and dose–response relationship of exercise-based interventions for muscle mass in older women with sarcopenia: a systematic review and network meta-analysis

**DOI:** 10.3389/fmed.2026.1938860

**Published:** 2026-08-25

**Authors:** Xinyi Gou, Junhui Zhu, Xinmiao Feng, Dongze Li, Qi Gao, Bin Ruan

**Affiliations:** 1 School of Sport Medicine and Rehabilitation, Beijing Sport University, Beijing, China; 2 Faculty of Education, Universiti Kebangsaan Malaysia, Bangi, Malaysia; 3 China Wushu College, Beijing Sport University, Beijing, China; 4 School of Sports Science and Engineering, East China University of Science and Technology, Shanghai, China

**Keywords:** dose–response relationships, exercise, female, network meta-analysis, non-pharmacological interventions, sarcopenia

## Abstract

**Background:**

Exercise and nutrition are the principal non-pharmacological strategies for sarcopenia, but the relative effects of different exercise combinations and the dose required to improve muscle mass in older women remain uncertain. This study compared exercise-based interventions and examined the dose-response relationship.

**Methods:**

PubMed, Web of Science, Embase, the Cochrane Library, and EBSCO were searched from inception to 1 July 2026. Eligible trials enrolled women aged ≥ 60 years female with sarcopenia. Risk of bias was assessed using RoB 2. A frequentist random-effects network meta-analysis combined direct and indirect evidence, with effects expressed as standardized mean differences (SMDs) and 95% confidence intervals (CIs). Interventions were ranked using P-scores. Network meta-regression examined control condition, age, intervention duration, weekly frequency, weekly exercise dose, and female proportion. A Bayesian model-based network meta-analysis assessed the dose–response relationship using MET-min/week.

**Results:**

Twenty-seven controlled trials involving 1,845 participants were included. Heterogeneity was moderate, with no significant global inconsistency. Most interventions improved muscle mass compared with control, although no significant effect was detected for Chinese traditional exercise. Resistance, aerobic, and balance training combined with nutritional supplementation produced the largest effect (SMD = 1.41, 95% CI 0.81–2.01) and ranked first (*P*-score = 0.882). Control condition, age, and weekly exercise dose were significant effect modifiers, but covariate adjustment did not materially alter the intervention rankings. The dose–response relationship was positive and nonlinear. Within the central evidence-supported range, the lower 95% credible limit first exceeded zero at 460 MET-min/week (SMD = 0.19, 95% CrI 0.01–0.37). At the upper boundary of this range (1,144 MET-min/week), the predicted SMD was 1.38 (95% CrI 1.08–1.61).

**Conclusion:**

Resistance-based multicomponent exercise may improve muscle mass in older women with sarcopenia, particularly when combined with nutritional supplementation. Exercise dose should be increased gradually according to individual tolerance. The reported dose values should be interpreted as evidence-supported estimates rather than fixed biological thresholds or universally optimal prescriptions.

**Systematic review registration:**

https://www.crd.york.ac.uk/PROSPERO/view/CRD420261449654, identifier: PROSPERO (CRD420261449654).

## 1 Introduction

Sarcopenia is a progressive, generalized skeletal muscle disorder primarily associated with aging (1). Although diagnostic thresholds vary across the major criteria, these frameworks converge on a common definition based on muscle strength, muscle mass, and physical performance (2, 3). Its global prevalence is estimated at 10–16% and is rising across regions (4, 5), with both prevalence and severity increasing with advancing age (6). As a geriatric syndrome, sarcopenia frequently coexists with obesity (7), frailty (8), and osteoporosis (9), thereby accelerating functional decline and clinical vulnerability. At the biological level, it is characterized by impaired muscle protein synthesis, diminished regenerative capacity, dysregulated anabolic signaling, and chronic low-grade inflammation (10, 11). When it coexists with obesity, this inflammatory state has been proposed to link sarcopenia with metabolic syndrome, type 2 diabetes, dyslipidemia, and cardiovascular disease (12, 13). These changes further raise the risks of falls, fractures, disability, cognitive impairment, and death, substantially compromising quality of life and imposing a considerable medical and socioeconomic burden (14–16).

This burden may differ between the sexes. Epidemiological studies have reported sex-related differences in the prevalence and risk-factor profile of sarcopenia (17, 18), although the direction and magnitude of these differences may depend on the diagnostic criteria, methods used to adjust muscle mass, age distribution, ethnicity, and population characteristics. Female sex has also been associated with sarcopenia risk in some cohort-based analyses (19). Older women generally have a lower baseline muscle reserve (20), while estrogen loss may reduce anabolic protection (21, 22) and contribute to changes in inflammatory status (23, 24). These factors may increase the risk of falls, fractures, and other adverse outcomes (25). Menopause may further contribute to an adverse cardiometabolic profile, including higher total and LDL cholesterol, lower HDL cholesterol, higher blood pressure, and insulin resistance (26, 27). These physiological and metabolic differences may also influence anabolic responses to exercise, exercise tolerance, and recovery, and may therefore modify the exercise dose required to improve muscle mass. Thus, evidence derived from mixed-sex populations may not fully reflect the effects or dose–response relationships of exercise in older women with sarcopenia.

Exercise and nutrition are the principal non-pharmacological strategies for managing sarcopenia (28). Evidence from systematic reviews and meta-analyses broadly indicates that resistance-based multicomponent programs, particularly when combined with nutritional supplementation, improve muscle strength, mass, physical function, and quality of life in older adults with sarcopenia (15, 29–33). These findings are nonetheless inconsistent, likely reflecting differences in population characteristics and intervention protocols.

Given the sex-related differences in sarcopenia (18, 34), specific evidence is needed, yet available female-focused meta-analyses have several limitations. First, the participants included across these reviews are potentially heterogeneous. Several reviews did not base their inclusion criteria on established diagnostic definitions of sarcopenia and enrolled predominantly postmenopausal women (35, 36), which weakens the specificity of their conclusions. Second, a recent female-focused network meta-analysis, though it integrates direct and indirect evidence to rank interventions, remains susceptible to heterogeneity in the definition of interventions (37). All exercise interventions were pooled under a single “exercise” node, without distinguishing specific modalities or separating single-component from multi-component programs; such coarse classification may introduce within-node heterogeneity and obscure the contribution of specific exercise components. Third, these analyses overlooked the covariate effects of age, control-group condition, and exercise-prescription variables, thereby hindering the design of individualized rehabilitation. Prior reviews have chiefly assessed which interventions are effective and which rank highest (37, 38), without examining the relationship between exercise dose and treatment effect (39). Muscle mass is both a core diagnostic indicator of sarcopenia and a principal target of non-pharmacological treatment (40). Addressing these gaps, the present study performed a network meta-analysis together with a dose-response analysis, to identify the most effective exercise components and estimate an effective weekly dose range for improving muscle mass in older women with sarcopenia.

## 2 Method

This systematic review and network meta-analysis followed the Cochrane Handbook and was reported according to the PRISMA 2020 statement. The protocol was registered in advance in PROSPERO (CRD420261449654).

### 2.1 Eligibility criteria

Eligibility followed the PICOS framework. Population: older women aged ≥60 years with sarcopenia diagnosed by EWGSOP/EWGSOP2, AWGS, IWGS, FNIH, SDOC, or comparable criteria. A trial was eligible if all participants were women or, in mixed-sex trials, if women constituted at least 70% of the enrolled sample; this ≥70% threshold defined a predominantly female trial. Participants who also presented with one related condition—obesity, osteoporosis, or frailty—remained eligible, provided that no more than one such condition was present. Intervention: structured exercise programs, including resistance, aerobic, balance, flexibility, and multi-component exercise, traditional Chinese exercises (CTX; Tai Chi, Baduanjin, Yijinjing, or comparable mind-body forms), and vibration training. These could be delivered in supervised, home-based, community-based, clinical, or mixed settings, alone or with non-pharmacological nutritional support. Comparator: usual or routine care, health education, waiting-list control, or maintenance of habitual lifestyle or physical activity, without a comparable structured exercise program. Outcomes: any measure related to muscle mass was considered eligible. Study design: only controlled trials were eligible, whether or not blinding of participants, personnel, or outcome assessors was reported.

Studies were excluded for any of the following reasons: sarcopenia was not defined by clear or established criteria; sarcopenic participants could not be separated from non-sarcopenic, borderline, or at-risk participants; women comprised fewer than 70% of participants, or the sex distribution was not reported; the exercise protocol was insufficiently described; outcome data were insufficient for effect-size calculation; or the article was not published in English.

### 2.2 Data sources and search

We searched PubMed, Web of Science, Embase, the Cochrane Library, and EBSCO from inception to 1 July 2026. The search terms covered three concepts: sarcopenia, exercise interventions, and Controlled trials. These were combined as (“sarcopenia” OR “muscle loss” OR “muscle weakness”) AND (“exercise” OR “training” OR “physical activity” OR “resistance training” OR “aerobic exercise” OR “balance training” OR “Tai Chi” OR “Baduanjin” OR “vibration training”) AND (“randomized controlled trial” OR “RCT” OR “CT” OR “controlled trial”). Full database-specific strategies are given in Supplementary material S1. This study also hand-searched the reference lists of included studies and relevant reviews.

### 2.3 Study selection

All records were imported into EndNote, and duplicates were removed before screening. Two reviewers (Xinmao and Qi) independently screened titles and abstracts against the eligibility criteria and discarded clearly irrelevant records. They then retrieved the full texts of the remaining records and assessed them independently. A reason was recorded for each full-text exclusion. Disagreements at either stage were resolved by discussion, and a third reviewer (Xinyi) was consulted when consensus could not be reached. The flow of records through the review is summarized in a PRISMA flow diagram.

### 2.4 Data extraction

Two reviewers independently extracted data from each study with a standardized, piloted form, and a third reviewer resolved any disagreement. For each study, we recorded the basic characteristics: first author, publication year, and study design. We also recorded the participant characteristics: mean age, the proportion of women, and sarcopenia diagnostic status, including the diagnostic criteria applied. The exact female proportion of each trial was recorded in the study-characteristics table, distinguishing all-female trials from predominantly female trials (70%−99% women), so that the influence of mixed-sex samples could be examined in the sensitivity and meta-regression analyses. We then extracted the intervention. This covered the exercise type, the intervention period, and the weekly frequency. It also covered the prescription itself—exercise selection, sets, repetitions, and rest intervals—together with the exercise intensity. Intensity was expressed in metabolic equivalents (METs). When it was not reported, two reviewers (Bin and Xinmiao) estimated it independently from the exercise type and protocol, averaged their estimates, and settled differences by discussion. This MET value was multiplied by session duration and weekly frequency to give the weekly MET dose (MET-min/week) used later in the dose-response analysis. Co-interventions, such as nutritional supplementation, were also recorded.

Outcomes were extracted in the same way and grouped into four domains. Muscle mass covered skeletal muscle quantity, size, or volume at the whole-body, appendicular, regional, or microscopic level. Eligible measures included whole-body and appendicular muscle mass indices, height- or weight-adjusted indices, regional muscle size, muscle volume, and cross-sectional area. These were obtained by validated body-composition, imaging, or histological methods, such as bioelectrical impedance analysis, dual-energy X-ray absorptiometry, ultrasound, computed tomography, magnetic resonance imaging, or muscle biopsy. Fat-free mass and other non-specific lean-mass indices were not treated as muscle mass. Measures of muscle quality, such as muscle density, attenuation, or intramuscular fat, were also excluded. When a study reported several eligible measures for the same domain, we kept them all as separate effect-size records rather than choosing one. Each record was linked to its parent study, so that measures from the same study could be treated as statistically dependent in the synthesis.

### 2.5 Quality assessment

Two reviewers independently classified each included study as randomized or non-randomized according to the reported allocation method, and a third reviewer resolved any disagreements. Risk of bias was assessed for the muscle-mass outcome. Randomized controlled trials were evaluated using the Cochrane Risk of Bias 2 (RoB 2) tool, covering bias arising from the randomization process, deviations from intended interventions, missing outcome data, measurement of the outcome, and selection of the reported result (41). Each randomized trial was judged as having low risk of bias, some concerns, or high risk of bias. Non-randomized controlled intervention studies were assessed using the Risk Of Bias In Non-randomized Studies of Interventions (ROBINS-I) tool, covering bias due to confounding, selection of participants, classification of interventions, deviations from intended interventions, missing data, measurement of outcomes, and selection of the reported result (42). Each non-randomized study was judged as having low, moderate, serious, or critical risk of bias.

### 2.6 Statistical analysis

#### 2.6.1 Network meta-analysis

Network meta-analyses were fitted in R using the netmeta package. A frequentist random-effects model combined direct and indirect evidence across the intervention categories, with the non-exercise control as the reference (43). As the included studies used different scales and instruments, effects were expressed as standardized mean differences (SMDs) with 95% confidence intervals (CIs). A network was analyzed for outcome domain when the data allowed. Network geometry was displayed as network plots, in which nodes represented intervention categories and edges represented direct comparisons, with node and edge sizes reflecting the available evidence (43). Pairwise effects were summarized in league tables, and effects relative to control were presented in forest plots. Interventions were ranked by *P*-scores, interpreted alongside the magnitude and precision of each estimate (44). Heterogeneity was quantified using τ^2^ and *I*^2^. Global inconsistency was assessed with the design-by-treatment interaction model, and local inconsistency by node-splitting (45, 46). Effect modification was examined by network meta-regression with a common covariate slope. The covariates were control type, mean age, intervention length, weekly frequency, weekly MET dose, and the proportion of female. For each covariate, the regression coefficient and its 95% CI (with the corresponding *p*-value) indicated whether it modified the intervention effect. Treatment effects relative to control were then re-estimated with each covariate fixed at its median or modal value, and the adjusted forest plots and rankings were compared with those of the primary analysis. Small-study effects were examined using comparison-adjusted funnel plots and the Egger and Begg tests when a sufficient number of studies was available (47, 48). Separate sensitivity analyses were conducted after excluding studies at high risk of bias and non-randomized controlled studies. A pre-specified sensitivity analysis also restricted the network meta-analysis to trials that enrolled women exclusively. The all-female analysis used the same intervention classification, reference group, and random-effects framework as the primary analysis. Effect estimates, heterogeneity, global and local inconsistency, *P*-scores, and intervention rankings were recalculated and compared with those from the full, predominantly female (≥70%) analysis. In addition, the proportion of women was retained as a covariate in the network meta-regression as a complementary assessment of effect modification rather than as a substitute for the all-female sensitivity analysis.

#### 2.6.2 Model-based dose-response network meta-analysis

The dose-response relationship was examined using a Bayesian random-effects model-based network meta-analysis with the MBNMAdose package. The dose variable was the weekly MET dose (MET-min/week) obtained during data extraction. Active exercise was modeled as a single agent scaled by dose. Effects were expressed as SMDs with 95% credible intervals (CrIs), with directions harmonized so that positive values indicated improvement. Before the functions were fitted, the dose network was displayed as a network plot with nodes defined by dose, and an unconstrained (split) network model was used to inspect the shape of the relationship (49). Four functions were then fitted: Emax, a restricted cubic spline (natural spline, three knots), a monotonically increasing nonparametric function, and an exponential function, each under common-effect and random-effects assumptions. The models were compared using the deviance information criterion (DIC), the effective number of parameters (pD), residual deviance, the between-study standard deviation, and convergence diagnostics (maximum R-hat and effective sample size) (50, 51). Two dose landmarks were derived from the best-fitting model within the range of doses actually observed, defined as the central 80% of observed doses (the 10th−90th percentiles). The lowest evidence-supported dose was the smallest dose in this range at which the posterior lower 95% credible limit exceeded zero, and the peak dose was the dose associated with the highest posterior median effect within the same range. Neither landmark was selected on the basis of nominal statistical significance, and the lowest evidence-supported dose was interpreted as a statistical evidence boundary rather than a biological minimum effective dose. Estimation used three Markov chain Monte Carlo chains, each with an initial 50,000 iterations, including 25,000 burn-in iterations, and a thinning interval of 10. Transitivity and consistency were considered before the estimates were interpreted (45, 46).

## 3 Results

### 3.1 Study selection

**Figure 1:**
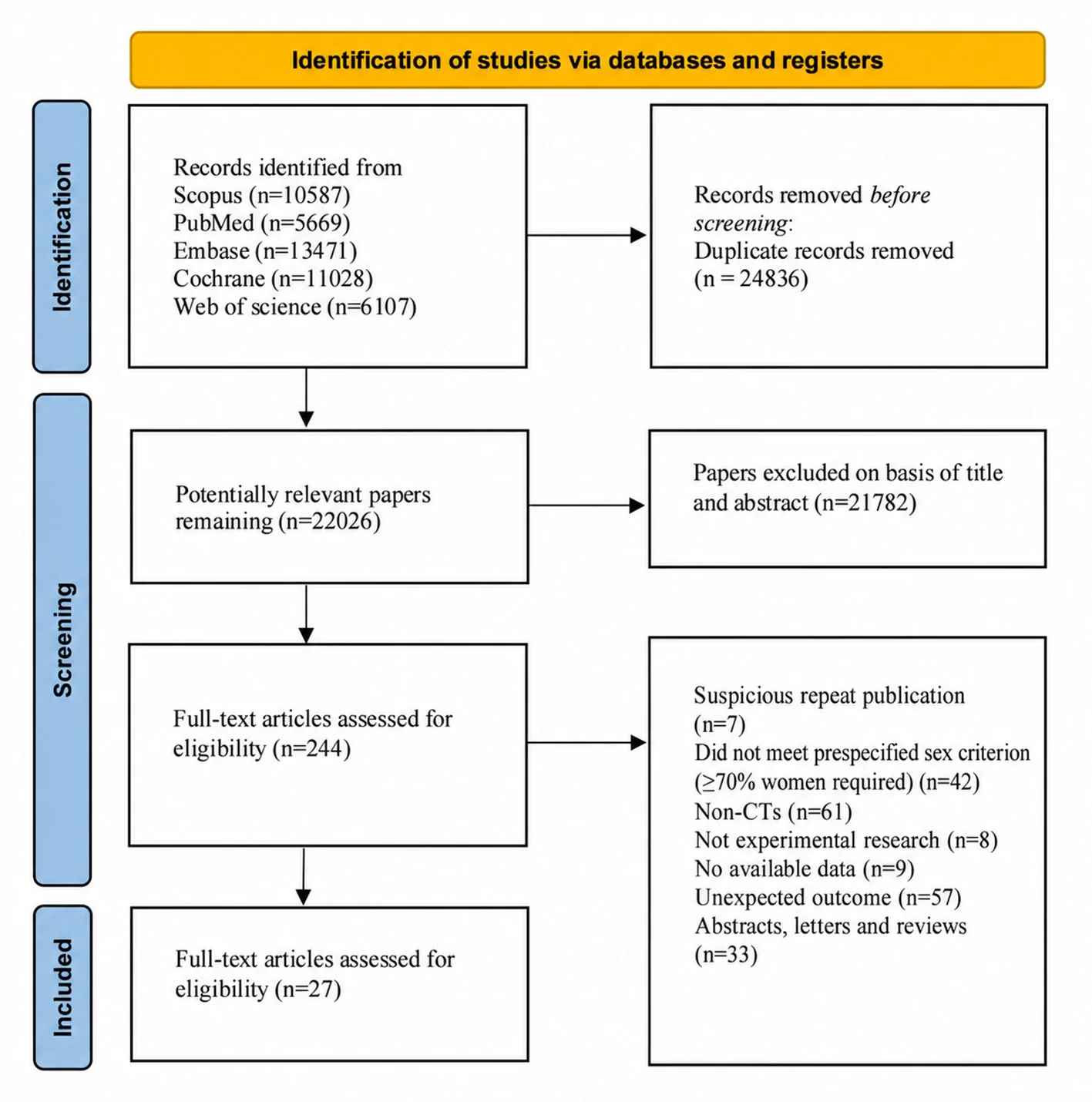
PRISMA flow diagram of the study selection process.

A total of 46,862 records were identified through searches of five electronic databases. After removing 24,836 duplicate records, 22,026 records remained for title and abstract screening, of which 21,782 were excluded. Subsequently, the full texts of 244 articles were assessed for eligibility. Of these, 217 articles were excluded because of suspected duplicate publication (*n* = 7), inclusion of non-sarcopenic populations (*n* = 42), failure to meet the prespecified proportion of women or inclusion of mixed-sex samples with < 70% women (*n* = 61), non-controlled trials (*n* = 8), non-experimental research (*n* = 9), unavailable data (*n* = 9), outcomes not relevant to this review (*n* = 57), or publication as abstracts, letters, or reviews (*n* = 33). Ultimately, 27 studies were included in the review (Figure 1). Of the 27 included controlled trials, 24 were randomized controlled trials and three were non-randomized controlled intervention studies.

### 3.2 Characteristics of included studies and quality assessment

The characteristics of the included studies are presented in Table 1 (further details in the Supplementary material S2). Across the 27 controlled trials, a total of 1,845 participants were included, of whom 1,137 received exercise or exercise-plus-nutrition interventions and 708 were assigned to control conditions. Resistance training alone (RT) was investigated in nine studies involving 209 participants; combined resistance and aerobic training (RAT) in seven studies involving 207 participants; combined resistance, aerobic, and balance training (RABT) in five studies involving 239 participants; combined resistance training and nutritional supplementation (RT + NS) in five studies involving 123 participants; combined resistance and aerobic training with nutritional supplementation (RAT + NS) in four studies involving 153 participants; Chinese traditional exercise (CTE) in two studies involving 47 participants; combined resistance, aerobic, and balance training with nutritional supplementation (RABT + NS) in two studies involving 36 participants; combined resistance and balance training with nutritional supplementation (RBT + NS) in two studies involving 45 participants; combined resistance and balance training (RBT) in one study involving 39 participants; combined resistance training and Chinese traditional exercise (RT + CTE) in one study involving 25 participants. A total of 855 participants across 27 studies received usual care, health education, maintenance of usual daily activities, or no exercise intervention; these conditions were collectively classified as the control group (CON). Detailed definitions of each intervention category are provided in Supplementary material S2. Because several trials included more than one eligible intervention arm, the numbers of studies across intervention categories were not mutually exclusive. The total sample size of the included studies ranged from 15 to 246 participants, with a median of 52 participants. Using the sample-size-weighted mean age across study arms, the study-level mean age ranged from 60.5 to 88.6 years, with a median of 71.9 years; the overall participant-weighted mean age was 72.6 years. The interventions lasted for 6 to 72 weeks, with a mean duration of 18.1 weeks, and were performed 2 to 5 times per week, with a mean frequency of 3.1 sessions per week. Weekly exercise dose ranged from 240 to 1,150 MET-min/week, with a mean of 571.3 MET-min/week and a median of 552.5 MET-min/week. Among the 27 included studies, six enrolled participants with an additional comorbid condition. Five studies included participants with sarcopenic obesity, whereas one study included participants with sarcopenia accompanied by frailty; the

**Table 1:** Characteristics of included studies.

References	Groups (sample size)	Sex and age	Training		
			Weeks	Frequency (sessions/weeks)	MET-min/week
Liao et al. (103)	RT: 33 CON: 23	RT: 66.67 ± 4.54 CON: 68.32 ± 6.05 100% female	12	3	630
Park et al. (104)	RAT: 25 CON: 25	RAT: 74.7 ± 5.1 CON: 74.7 ± 5.1 100% female	24	5	1225
Tsekoura et al. (105)	Group-Based RABT: 18 Home-based RABT: 18 CON: 18	Group-Based RABT: 74.56 ± 6.04 89% female Home-based RABT: 71.17 ± 6.47 84% female CON: 72.89 ± 8.31 89% female	12	3	945
Piastra et al. (106)	RT: 35 CON: 37	RT: 69.9 ± 2.7 CON: 70.0 ± 2.8 100% female	36	2	450
Huang et al. (107)	RT: 18 CON: 17	RT: 68.89 ± 4.91 CON: 69.53 ± 5.09 100% female	12	3	725
Zhu et al. (108)	RAT: 40 RAT + NS: 36 CON: 37	RAT: 74.5 ± 7.1, 72.5% female RAT + NS: 74.8 ± 6.9, 80.6% female CON: 72.2 ± 6.6, 78.4% female	24	3	945
Makizako et al. (109)	RABT: 36 CON: 36	RABT: 74.1 ± 6.6, 72.7% female CON: 75.8 ± 7.3, 71.4% female	12	6	1,055
Kim et al. (110)	RAT + NS: 36 RAT: 35 CON: 34	RAT + NS: 80.9 ± 4.2 RAT: 81.1 ± 4.3 CON: 81.1 ± 5.1 100% female	12	2	615
Shahar et al. (111)	RT + BT + AT + NS: 15 RT + BT + AT: 19 CON: 16	RT + BT + AT + NS: 65.20 ± 4.87 RT + BT + AT: 69.74 ± 5.46 CON: 67.25 ± 5.48 100% female	12	2	575
Kemmler et al. (112)	RT + BT + AT: 123 CON: 123	RT + BT + AT: 68.9 ± 3.9 CON: 69.2 ± 4.1 100% female	72	4	1,245
Kim et al. (113)	RBT +NS: 38 RBT: 39 CON: 39	RBT + NS: 79.5 ± 2.9 RBT: 79.0 ± 2.9 CON: 79.2 ± 2.8 100% female	12	2	575
Monti et al. (69)	RABT + NS: 21 CON: 24	RABT + NS: 79.6 ± 5.8 CON: 78.0 ± 6.1 100% female	–	5	1,025
Flor-Rufino et al. (114)	RT: 20 CON: 18	RT: 79.9 ± 7.2 CON: 79.6 ± 7.7 100% femlae	24	2	900
Seo et al. (115)	R T: 12 CON: 10	RT: 70.3 ± 5.38 CON: 72.9 ± 4.75 100% femlae	16	3	1,005
Jung et al. (116)	RT + AT: 13 CON: 13	RT + AT: 75.0 ± 3.9 CON: 74.9 ± 5.2 100% female	12	3	870
Morawin et al. (52)	CTE: 23 CON: 21	70.5 ± 5.8 90% female	40	2	360
Liu et al. (101)	RT + NS: 30 RT: 30	RT + NS: 66.07 ± 4.05 RT: 65.65 ± 4.60 100% female	12	3	1,125
Caldo-Silva et al. (117)	RT + NS: 8 RBT + NS: 7 CON: 13	RT + NS: 80 ± 6.1 RBT + NS: 86.7 ± 4 CON: 83.1 ± 5.4 100% female	16	2	RT + NS: 675 RBT + NS: 815
Jung et al. (102)	RAT: 14 CON: 14	RAT: 78.14 ± 3.72 CON: 78.21 ± 3.72 100% female	12	3	1,125
da Cruz Alves et al. (70)	RT + NS: 17 RT: 15	RT + NS: 70.6 ± 3.94 RT: 71.4 ± 6.21 100% female	14	3	635
Osuka et al. (118)	RT + NS: 39 RT: 39 CON: 39	RT + NS: 73.5 ± 4.2 RT: 71.8 ± 4.1 CON: 71.6 ± 4.2 100% female	12	3	595
Hamaguchi et al. (119)	RT: 7 CON: 8	RT: 60.4 ± 2.7 CON: 60.6 ± 2.3 100% female	6	2	720
Magtouf et al. (120)	RABT: 25 CON: 25	RABT: 75.9 ± 5.4 CON: 76.3 ± 5.4 100 % female	16	3	835
Yuenyongchaiwat and Akekawatchai (121)	RAT: 30 CON: 30	69.23 ± 6.71 73.33% female	12	5	1,115
Chen et al. (88)	CTE + RT: 25 CON: 24	CTE + RT: 65.68 ± 2.5 CON: 65.21 ± 2.6 100% female	8	3	825
Wang et al. (122)	RAT + NS: 50 RAT: 50 CON: 51	RAT + NS: 70.16 ± 4.32 84% female RAT: 69.72 ± 3.60 80% female CON: 69.88 ± 3.29 86.3% female	12	3	1,075
Thavonlun et al. (123)	RT + NS: 29 RAT + NS: 31	RT + NS: 68.6 ± 3.7 RAT + NS: 68.0 ± 4.0 73.4% female	24	5	RT + NS: 625 RAT + NS: 825

AT, aerobic training; BT, balance training; CON, control group; CTE, Chinese traditional exercise; NS, nutritional supplementation; RAT, resistance and aerobic training; RABT, resistance, aerobic, and balance training; RBT, resistance and balance training; RT, resistance training.

remaining 21 studies enrolled participants without a specified comorbid condition.

The risk of bias was assessed separately according to study design, with muscle mass specified as the outcome of interest. The 24 randomized controlled trials were evaluated using the Cochrane RoB 2 tool. Of these, two studies (8.3%) were judged to have a low overall risk of bias, 21 (87.5%) raised some concerns, and one study (4.2%), conducted by Morawin et al., was judged to have a high overall risk of bias (52). Most randomized trials assessed muscle mass using relatively objective and consistently applied methods, such as dual-energy X-ray absorptiometry (DXA) or bioelectrical impedance analysis (BIA); therefore, the risk of bias in the outcome measurement domain was generally low. The main concerns arose from insufficient reporting of random-sequence generation and allocation concealment, incomplete descriptions of participant attrition and the handling of missing outcome data, and the absence of verifiable preregistered protocols or prespecified primary outcomes. In Morawin et al., only 44 of the 80 randomized participants completed the study, resulting in a high overall risk of bias. The three non-randomized controlled studies were assessed using the ROBINS-I tool. Two studies were judged to have a serious overall risk of bias, whereas one was judged to have a moderate overall risk. The principal concerns related to confounding arising from non-random or location-based allocation, selection of participants into the analyzed sample, deviations from the intended interventions, and incomplete outcome data. Intervention classification was generally judged to be at low risk because the intervention groups were clearly defined, whereas the risk associated with outcome measurement varied according to the measurement method and reporting of assessor blinding. Overall, the randomized evidence predominantly raised some concerns, while the non-randomized evidence was subject to greater methodological limitations and should therefore be interpreted with additional caution. Detailed risk-of-bias assessments are presented in Supplementary material S3.

### 3.3 Network meta-analysis and dose-response relationships

**Figure 2:**
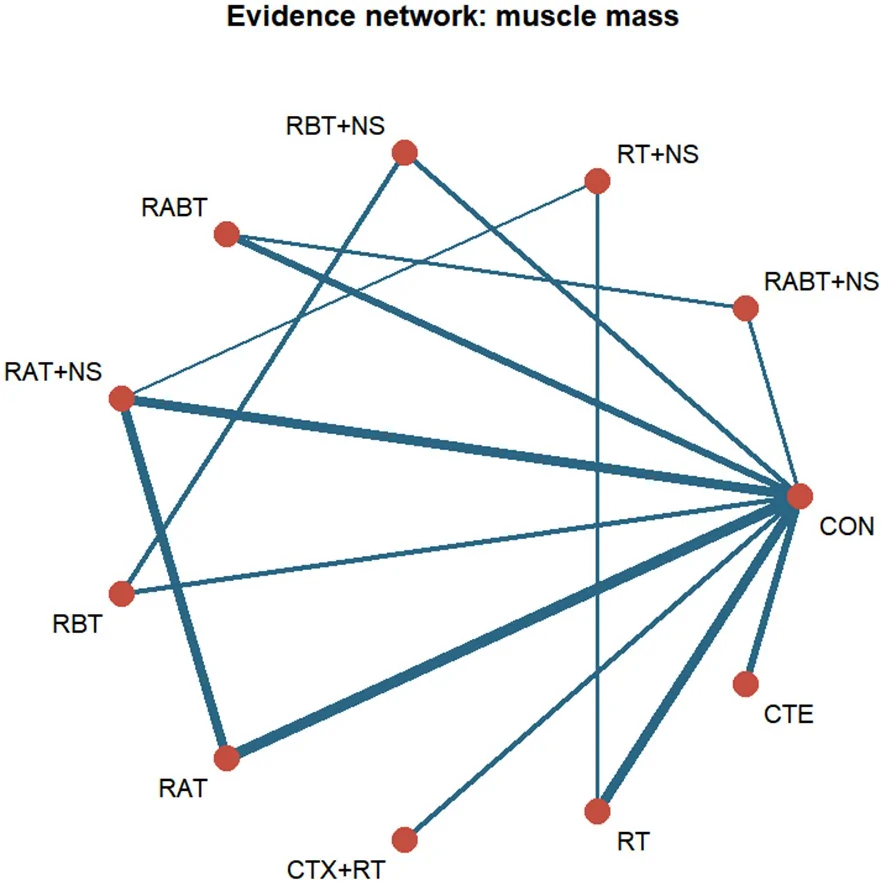
Network plot of direct comparisons among interventions for muscle mass. Nodes represent intervention groups, connecting lines represent available direct comparisons, and line thickness reflects the relative amount of direct evidence. AT, aerobic training; BT, balance training; CON, control group; CTE, Chinese traditional exercise; NS, nutritional supplementation; RAT, resistance and aerobic training; RABT, resistance, aerobic, and balance training; RBT, resistance and balance training; RT, resistance training.

**Figure 3:**
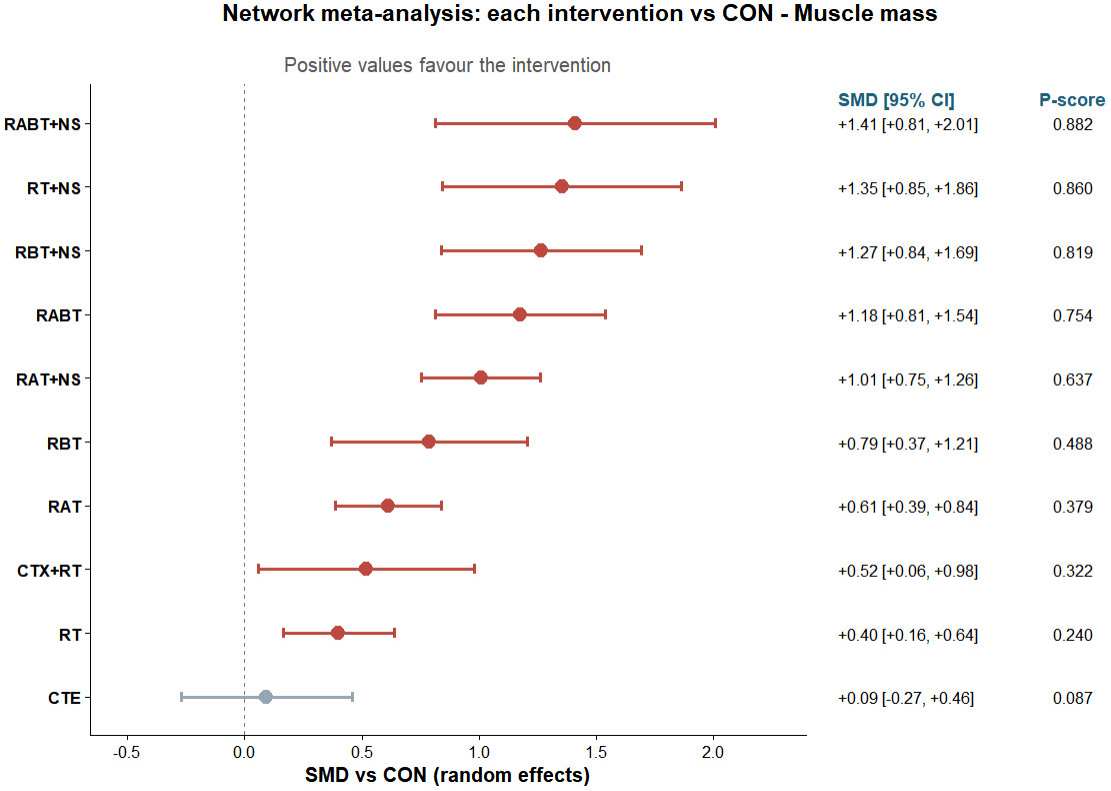
Forest plot of the network meta-analysis comparing each intervention with the control group for muscle mass. Points represent standardized mean differences (SMDs), horizontal lines represent 95% confidence intervals (CIs), and positive values favor the intervention. *P*-scores indicate the relative ranking of interventions. AT, aerobic training; BT, balance training; CON, control group; CTE, Chinese traditional exercise; NS, nutritional supplementation; RAT, resistance and aerobic training; RABT, resistance, aerobic, and balance training; RBT, resistance and balance training; RT, resistance training.

**Figure 4:**
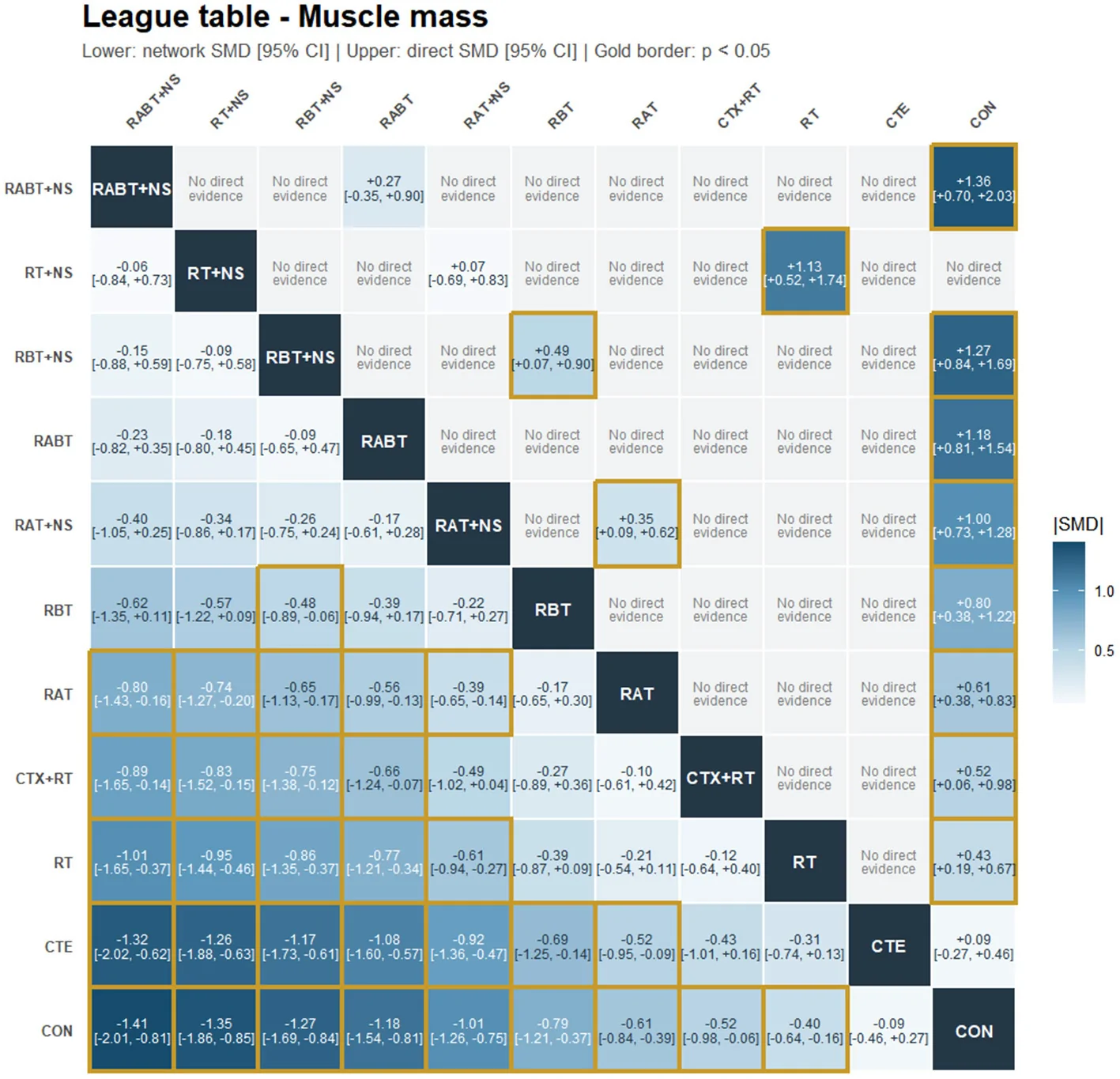
League table summarizing network and direct comparisons for muscle mass. The lower triangle presents network SMDs with 95% CIs, whereas the upper triangle presents direct SMDs with 95% CIs. Gold borders denote statistically significant comparisons (*p* < 0.05), and color intensity reflects the absolute effect size. AT, aerobic training; BT, balance training; CON, control group; CTE, Chinese traditional exercise; NS, nutritional supplementation; RAT, resistance and aerobic training; RABT, resistance, aerobic, and balance training; RBT, resistance and balance training; RT, resistance training.

Heterogeneity results showed moderate (*I*^2^ = 48.8%, Supplementary 4). The results of design-by-treatment interaction test showed that global inconsistency was not significant (*P* = 0.6777, Supplementary material S4). The SIDE test showed that the percentage of comparisons with evidence of inconsistency was 3.8% (Supplementary material S4). Additionally, the comparison-adjusted funnel plot had good symmetry for all outcomes, and the results of Egger's test (*P* < 0.05) showed that no small study effect was found (Supplementary material S5). Figure 2 showed the direct comparison and sample size distribution between the types for muscle mass. Compared with CON, all interventions except CTE significantly improved muscle mass, with SMDs (95% CIs) ranging from 0.40 (0.16 to 0.64) for RT to 1.41 (0.81 to 2.01) for RABT + NS (Figure 3). The network pairwise comparisons showed that RABT + NS was significantly more effective than RAT, CTX + RT, RT, and CTE (Figure 4). RABT + NS also ranked first, with a *P*-score of 0.882, followed by RT + NS (*P*-score = 0.860) and RBT + NS (*P*-score = 0.819). In addition, RABT + NS, RT + NS, RBT + NS, RABT, and RAT + NS all showed large effect sizes (SMD > 0.8). By contrast, CTE did not significantly improve muscle mass compared with CON (SMD = 0.09, 95% CI: −0.27 to 0.46). Meta-regression analysis indicated that the control condition, participant age, and weekly exercise dose expressed as MET-min/week were significant effect modifiers (Supplementary material S6). In contrast, intervention duration (*P* = 0.152), weekly exercise frequency (*P* = 0.931), and the proportion of female participants (*P* = 0.823) were not significantly associated with the estimated treatment effects. After fixing the significant covariates at the prespecified values—sarcopenia education as the control condition, a mean age of 74.5 years, and an exercise dose of 797.5 MET-min/week—the ordering of interventions in the ranking plot remained unchanged. This finding suggests that the relative intervention hierarchy was robust to adjustment for these covariates. Sensitivity analyses were conducted after excluding studies at high risk of bias, non-randomized controlled studies, studies that did not exclusively enroll women, and studies involving participants with comorbid conditions. Across these analyses, the intervention rankings remained broadly unchanged, and resistance-based multicomponent exercise combined with nutritional supplementation remained relatively highly ranked. When the analysis was restricted to studies enrolling women exclusively, heterogeneity decreased to a low level. Detailed results are presented in Supplementary material S6.

**Figure 5:**
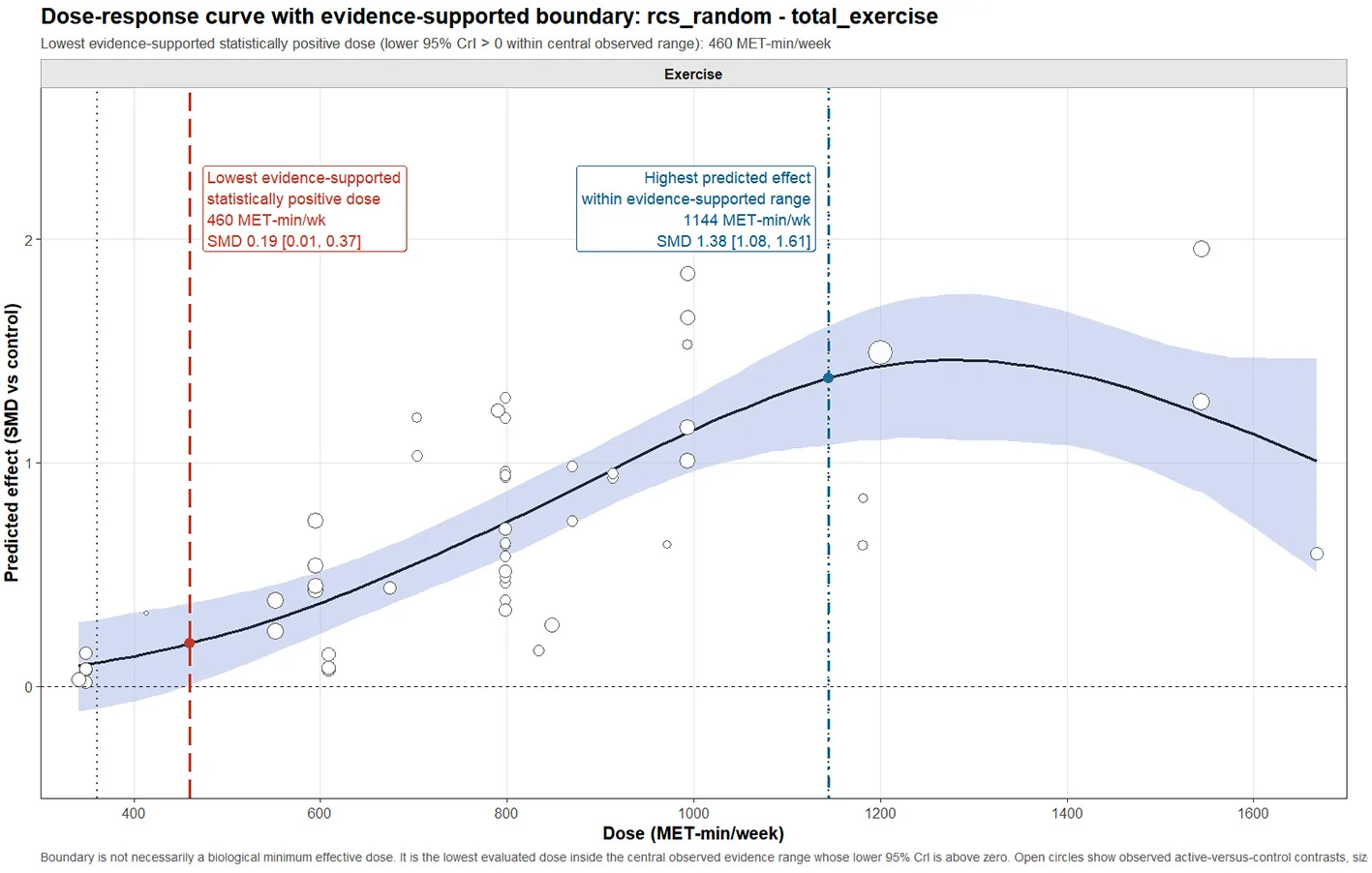
Dose–response association between total weekly exercise dose and muscle mass. The solid curve shows the predicted SMD vs. control, the shaded band shows the 95% credible interval (CrI), and open circles show observed active-vs.-control contrasts. Vertical dashed lines identify the lowest evidence-supported statistically positive dose (400 MET-min/week) and the dose associated with the highest predicted effect within the evidence-supported range (1,106 MET-min/week).

Given the anticipated between-study heterogeneity, model selection focused primarily on the random-effects dose–response functions. Based on model fit, complexity, and convergence, the three-knot random-effects restricted cubic spline model was selected for the dose–response analysis. Model selection considered the DIC, pD, posterior deviance, residual deviance, between-study standard deviation, R-hat values, and effective sample sizes. Supplementary material S8 reports the supporting analyses, including the dose-specific evidence network, unconstrained split-dose estimates, comparisons of candidate dose–response functions, the observed dose distribution, spline knot locations, convergence diagnostics, dose-specific posterior estimates, and assessments of consistency and transitivity. The selected model showed acceptable convergence and adequate fit, supporting its use to estimate the dose–response relationship within the observed evidence range. The results indicated a nonlinear positive association between weekly exercise dose and improvement in muscle mass (Figure 5). Within the central evidence-supported range of the observed data, 460 MET-min/week was the lowest evaluated dose at which the lower bound of the 95% credible interval exceeded zero, with an estimated SMD of 0.19 (95% CrI: 0.01–0.37). At the upper boundary of this range (1,144 MET-min/week), the predicted SMD was 1.38 (95% CrI: 1.08–1.61). These values represent statistical landmarks supported by the available evidence and should not be interpreted as biological thresholds or an optimal exercise prescription. The predicted effect increased across this range, reaching its highest value at 1,106 MET-min/week, with an estimated SMD of 1.51 (95% CrI: 1.27 to 1.71). These values reflect the distribution and evidential support of the available data and should not be interpreted as definitive biological thresholds, a minimum effective dose, or an optimal exercise dose. Estimates outside the central evidence-supported range were less well supported by the available data and should be interpreted cautiously.

## 4 Discussion

A total of 27 controlled trials were included in this study. The pooled analysis showed that, compared with single-mode exercise interventions, resistance training-based multicomponent exercise programs produced greater improvements in muscle mass among older women with sarcopenia. The intervention effect was further enhanced when exercise was combined with nutritional supplementation. Unlike most previous meta-analyses, which primarily examined overall intervention effects or the relative ranking of competing interventions, the present study also identified age, control-group type, intervention duration, and weekly exercise dose (MET-min/week) as potential effect modifiers. Specifically, the intervention effect decreased with increasing participant age. When the control group received health education, the relative effect of exercise was smaller than when the comparator was maintenance of usual daily activities. Weekly exercise dose was positively associated with the magnitude of improvement in muscle mass. The dose–response analysis identified 460 MET-min/week as the lowest evaluated dose within the central evidence-supported range at which the lower 95% credible interval exceeded zero. The predicted effect continued to increase across this range, whose upper boundary was 1,144 MET-min/week.

Consistent with previous meta-analyses of older populations comprising both sexes, the present study showed that nonpharmacological interventions centered on exercise and nutrition can improve muscle mass in women with sarcopenia (30, 53–55). The combined direct and indirect evidence indicated that resistance training-based multicomponent exercise programs generally produced greater improvements in muscle mass than single-mode exercise interventions (31, 37, 56), and that this advantage might be further enhanced by nutritional supplementation (56–58). Unlike previous studies that broadly classified exercise modalities as either “exercise” or “multicomponent training” (37), the present study explicitly identified individual exercise components, including resistance, aerobic, and balance training, thereby improving the comparability of different intervention programs. Resistance training may be the principal component responsible for muscle hypertrophy, whereas the benefits of balance and aerobic training may be reflected more specifically in fall prevention (59, 60), physical performance (33, 61), and body composition (62, 63), although these outcomes were not directly assessed in the present study. Overall, the available evidence provides greater support for appropriately integrating other exercise components into resistance-based training programs. The heterogeneity of exercise prescriptions for older adults should also be considered. In the subgroup analysis (64), no significant differences in lean body mass or leg strength were observed between combined interventions and exercise-only interventions among women. This discrepancy may be attributable, at least in part, to the physiological characteristics of postmenopausal women (34, 65). Inadequate protein intake may be more prevalent among women than among men (66), while hormonal changes during menopause may impair protein metabolism and reduce anabolic efficiency (21). Preclinical models have further suggested that estrogen deficiency during menopause may reduce skeletal muscle sensitivity to anabolic stimuli, a phenomenon known as “anabolic resistance” (67). These sex-related differences highlight the potential importance of protein supplementation in the management of sarcopenia in women (68). In addition to protein, two studies included in the present analysis provided vitamin D (69) and fish oil (70) supplementation. However, because only a limited number of nutrition-related randomized controlled trials were included, the present study could not determine whether the benefits differed among nutrients or across supplementation doses (71).

Although resistance training is regarded as the primary exercise modality for increasing muscle mass in individuals with sarcopenia (30, 53, 72), several studies have reported that resistance training alone does not improve muscle mass in this population (39, 62, 73, 74). One possible explanation for these inconsistent findings is variation in the sources and number of included studies, particularly whether Chinese-language studies were eligible for inclusion. Ran et al. further suggested that the absence of a significant improvement in muscle mass might be related to the lack of protein supplementation (74). Discrepancies among studies may also arise from differences in training intensity, frequency, and total training volume. Fu et al. reported that low- to moderate-load resistance training was sufficient to improve muscle mass (72). Hernandez-Martinez et al. (54) indicated that progressive resistance training could improve muscle strength and increase muscle mass in older adults by promoting protein synthesis and activating key molecular signaling pathways involved in muscle-fiber metabolism and function, including the PI3K-Akt-mTOR pathway (68, 75). Although Yan et al. (39) did not specifically discuss interventions that failed to produce beneficial effects, their findings suggested that training volume, rather than training frequency (76), may be more relevant to muscle hypertrophy. The meta-regression in the present network meta-analysis similarly indicated that MET-min/week, which represents total weekly exercise volume, was significantly associated with the intervention effect. Previous research has suggested a U-shaped dose-response relationship between training volume and skeletal muscle hypertrophy (77). A recent review by Bernárdez-Vázquez et al. (78) reported a similar pattern and recommended at least 10 sets per muscle group per week to optimize muscle hypertrophy. Accordingly, an adequate training volume may be a key variable contributing to muscle hypertrophy in women with sarcopenia (79). Resistance-training intensity appears to have a more pronounced influence on strength gains (80); however, some studies have indicated that, when total training volume is matched, differences in intensity do not significantly affect muscle hypertrophy (81). High-energy phosphate stores in skeletal muscle have been reported to decline after menopause, which may limit women's capacity to perform high-intensity exercise (82, 83). This physiological characteristic suggests that low- to moderate-intensity resistance training performed with an adequate training volume may be more appropriate for women with sarcopenia.

The present network meta-analysis also included vibration training and Chinese traditional exercise (CTE) as single-modality interventions. In contrast to previous studies, the present analysis did not detect a statistically significant improvement in muscle mass for either modality (56, 84). For CTE, this finding should be interpreted cautiously because the node was supported by only a small number of studies and participants, resulting in imprecise estimates. In addition, the exclusion of Chinese-language publications limited the coverage of the available evidence on CTE. A further limitation is that CTE encompasses several distinct forms of mind-body exercise, including qigong (84), Baduanjin (85), Wuqinxi (86), and tai chi (87), and the available evidence did not allow their specific effects to be distinguished. One study reported a beneficial effect of CTE combined with resistance training (88); however, this isolated finding should be considered preliminary and is insufficient to determine whether adding resistance training modifies the effect of CTE on muscle mass.

Meta-regression indicated that age, intervention duration, and control-group conditions might significantly modify the intervention effects on muscle mass. Age was negatively associated with the intervention effect, suggesting that muscular adaptations to exercise stimuli gradually diminish with advancing age among older women with sarcopenia. This finding was consistent with the meta-regression results reported by Delaire et al. (89), Sánchez et al. (64) and Cao et al. (56), in which age was identified as a significant moderator of the intervention effect. In the analysis by Delaire et al. (89), participants in studies classified as having “ineffective interventions” were substantially older than those in studies classified as having “effective interventions.” Their model further showed that each 1-year increase in age was associated with an average reduction of approximately 0.06 in the effect size, and this negative association remained significant after robust variance estimation. The age-related attenuation of the intervention effect may be primarily attributable to the progressive development of anabolic resistance with aging (90). Existing evidence indicates that the anabolic response of skeletal muscle to resistance training begins to decline at approximately 60 years of age and becomes more limited among adults aged 75 years or older (90, 91). A meta-analysis of participants with a mean age of approximately 80 years found no significant effect of resistance training on appendicular muscle mass (89). Potential mechanisms include attenuation of exercise- and amino acid-induced muscle protein synthesis (MPS) with advanced age (92), REDD1-mediated inhibition of the mTORC1 pathway (93), and dysregulated microRNA transcriptional control (94). Reduced intramuscular amino acid availability, satellite-cell dysfunction, and delayed tissue repair may further constrain muscle-fiber hypertrophy and ultimately diminish training-induced gains in muscle mass (95). Therefore, for older women with sarcopenia at an advanced age, priority should be given to correct exercise technique, adherence, and safety, followed by gradual increases in training load according to individual tolerance. Given the delayed post-exercise recovery commonly observed at advanced ages (96), longer recovery intervals should be scheduled between training sessions. Accordingly, strategies to maintain adherence among older women with sarcopenia warrant particular attention.

Control-group conditions were also associated with the estimated intervention effects. Compared with control groups instructed only to maintain their usual daily activities, health-education controls were associated with smaller relative effects of exercise. One possible explanation is that health education may improve exercise awareness, dietary behaviors, or habitual physical activity, thereby providing some benefit to control participants and reducing between-group differences (97, 98). However, this interpretation remains hypothetical because direct empirical evidence was lacking. The educational interventions also varied across studies: some provided a single session at the beginning of the intervention, whereas others included regular follow-up sessions with individualized health advice. This variation may have contributed to heterogeneity among health-education control conditions, although its influence on the estimated effects could not be determined from the available data (99). Although intervention duration was not a significant effect modifier in the present meta-regression, the duration of an exercise program may still influence adherence at the individual-study level. Wu et al. reported that interventions lasting longer than 24 weeks were associated with reduced adherence among individuals with sarcopenia (100). This finding suggests that program duration should be considered primarily in relation to feasibility and adherence rather than as an established modifier of the treatment effect.

Meta-regression indicated that weekly exercise volume, expressed as MET-min/week, was an important effect modifier of improvements in muscle mass among older women with sarcopenia. Restricted cubic spline analysis further indicated a positive, nonlinear dose-response association. Within the central evidence-supported range, 460 MET-min/week was the lowest evaluated dose at which the lower 95% credible interval exceeded zero, whereas 1,144 MET-min/week defined the upper boundary of this range. These values reflect the distribution and statistical support of the available evidence and should not be interpreted as a biological minimum effective dose, a true maximum, or an optimal exercise prescription. The dose range identified in this study was broadly comparable to that reported by Yan et al. for improving muscle strength in older adults with sarcopenia (39). However, the dose estimates from the present study should be interpreted cautiously because the analysis was restricted to data within the central 80% of the observed dose distribution. MET-min reflects the combined exposure resulting from exercise intensity, frequency, and duration. Given the physiological characteristics and exercise tolerance of older women with sarcopenia, the present findings support a graded and individualized dose-progression strategy. Weekly MET-min should be increased progressively, primarily by increasing resistance-training volume when the goal is to improve muscle mass. Large increases within a single progression stage should be avoided, and adequate recovery should be allowed before the same muscle groups are exposed to another higher-load session. This individualized process requires regular follow-up by qualified professionals. Some of the included studies increased exercise volume in a stepwise manner at intervals of 2–4 weeks (101, 102). During the initial phase, resistance training may begin with elastic-band or body-weight exercises to permit a lower initial load and facilitate familiarization with correct movement patterns (53). Resistance and range of motion should be adjusted to each participant's baseline functional capacity and then progressively increased as technique and exercise tolerance improve. Higher-load sessions targeting the same muscle groups should generally be scheduled on nonconsecutive days, and the rate of progression should be modified according to recovery and individual tolerance.

## 5 Limitations

Several limitations should be considered. First, a small number of studies included mixed-sex samples, although women accounted for more than 70% of participants. Adjusting the proportion of women to the median value did not materially change the intervention rankings, suggesting a limited influence on the main findings. Although the dose-response analysis was not repeated in the all-female dataset, the included evidence predominantly represented women. Therefore, the estimated dose-response relationship is expected to primarily reflect responses in older women, although future validation using exclusively female datasets would further strengthen its sex-specific applicability. Second, some studies included in the dose-response analysis combined exercise with nutritional supplementation, which may have influenced the estimated dose range. Therefore, the estimated range may be more applicable in the context of adequate nutritional support. Third, several included studies had methodological limitations, which may reduce confidence in the estimates. Fourth, some intervention nodes were supported by relatively few studies and participants, which may have reduced the precision and stability of the effect estimates and treatment rankings. *P*-scores should therefore be interpreted as relative rankings within the available evidence network rather than definitive evidence of comparative superiority, particularly for sparsely informed interventions. Fifth, the model-derived dose landmarks provide quantitative, evidence-informed reference points for exercise prescription in older women with sarcopenia. Their interpretation should nevertheless consider the range of the available evidence and individual factors such as health status, physical capacity, exercise tolerance, and nutritional support, rather than treating them as fixed universal targets. Finally, variations in resistance type, intensity, repetitions, progression, and equipment prevented standardized analyses of individual exercise-prescription variables. Similar heterogeneity was observed in aerobic and balance training.

## 6 Conclusion

Resistance-based multicomponent exercise may improve muscle mass in older women with sarcopenia. Programs incorporating more diverse exercise components and nutritional supplementation may provide greater benefits. Within individual tolerance, a progressively increased weekly exercise dose may produce greater gains in muscle mass. Participant age and the type of control condition may also modify the intervention effects. Healthcare professionals may use these findings to individualize exercise components, weekly dose, and nutritional support according to each patient's age, health status, functional capacity, and exercise tolerance.

## Data Availability

The original contributions presented in the study are included in the article/Supplementary material, further inquiries can be directed to the corresponding authors.
