# Correction: Comparative effectiveness and dose–response relationship of exercise-based interventions for muscle mass in older women with sarcopenia: a systematic review and network meta-analysis

**DOI:** 10.3389/fmed.2026.1991448

**Published:** 2026-09-25

**Authors:** Xinyi Gou, Junhui Zhu, Xinmiao Feng, Dongze Li, Qi Gao, Bin Ruan

**Affiliations:** 1 School of Sport Medicine and Rehabilitation, Beijing Sport University, Beijing, China; 2 Faculty of Education, Universiti Kebangsaan Malaysia, Bangi, Malaysia; 3 China Wushu College, Beijing Sport University, Beijing, China; 4 School of Sports Science and Engineering, East China University of Science and Technology, Shanghai, China

**Keywords:** dose–response relationships, exercise, female, network meta-analysis, non-pharmacological interventions, sarcopenia

The funding statement was incorrectly published as:

The author(s) declared that financial support was not received for this work and/or its publication.

The corrected funding statement reads:

The author(s) declared that financial support was received for this work and/or its publication. This research was supported by a grant from the Fundamental Research Funds for the Central Universities (Exercise Rehabilitation Science Laboratory), Grant No. **2025KYPT12**.

The original article has been updated.

